# Family planning and preimplantation testing: family experiences in congenital adrenal hyperplasia

**DOI:** 10.3389/fendo.2024.1482902

**Published:** 2025-01-07

**Authors:** Jessica L. Sandy, Grant Betts, Jessica L. Harper, Suzanne M. Nevin, Rebecca Deans, Kristen A. Neville

**Affiliations:** ^1^ Department of Endocrinology, Sydney Children’s Hospital, Randwick, NSW, Australia; ^2^ Children’s Hospital Westmead Clinical School, University of Sydney, Sydney, NSW, Australia; ^3^ Institute of Endocrinology and Diabetes, The Children’s Hospital at Westmead, Westmead, NSW, Australia; ^4^ School of Clinical Medicine, University of New South Wales Sydney, Sydney, NSW, Australia; ^5^ Behavioural Sciences Unit, Kids Cancer Centre, Sydney Children’s Hospital, Randwick, NSW, Australia; ^6^ Department of Gynaecology, Royal Hospital for Women, Randwick, NSW, Australia

**Keywords:** congenital adrenal hyperplasia, preimplantation diagnosis, decision-making, prenatal diagnosis, family planning

## Abstract

**Introduction:**

Pre-implantation testing (PGT) is often suggested by healthcare professionals (HCP) to parents of children with congenital adrenal hyperplasia (CAH) considering subsequent children. Despite this, some families choose to conceive naturally without genetic testing and intervention. The aims of this study were to explore fertility choices of couples with a child with CAH and the decision making process and perceptions behind these choices, and to explore the families’ lived experiences with CAH and the couples’ subsequent fertility journey. A better healthcare professional understanding of these experiences may subsequently help guide clinicians to better manage and support families of children with CAH and other autosomal recessive conditions.

**Methods:**

All parents of current children of a tertiary service in 2020 with 21-hydroxylase deficient CAH who made an active decision regarding family planning after diagnosis of their index child were invited to participate in a semi-structured interview. Thematic analysis was performed using an inductive, semantic approach.

**Results:**

Thirty families (34 children) were identified. Fourteen considered subsequent children and had directed genetic counselling. Eight decided to have additional children of whom seven agreed to participate. Thematic analysis identified six key domains. Psychological impact surrounding the CAH diagnosis was long-lasting, causing symptoms of trauma including depression and anxiety, and influencing a couple’s choice to pursue PGT to avoid having another affected child. The perception of the index child having a mild phenotype, and fear of a more severe phenotype, often supported this decision. Conversely, lived experience of CAH and low day-to-day impact, along with a negative experience of PGT, with a greater than anticipated financial, physical, and emotional toll, led some families to subsequently consider natural conception. The role of the healthcare professional (HCP) was important in the CAH and family planning journeys. A perceived poor understanding of CAH, overstating its potential seriousness, contributed to distress. Parents reported feeling pressured to undergo PGT. Peer-support had a universally positive impact on family experience.

**Discussion/conclusions:**

This study highlights the complex and dynamic nature of fertility decision-making, and the importance of HCP empathy and open-mindedness. Education of HCP and encouraging peer support may improve the CAH and fertility journey for families.

## 1 Introduction

Congenital adrenal hyperplasia (CAH) due to 21-hydroxylase deficiency is a genetic, chronic, potentially life-threatening condition that affects adrenal gland production of glucocorticoids, mineralocorticoids and androgens. Classic (severe) CAH presents in infancy, with chromosomal female (46,XX) infants often presenting with ambiguous genitalia at birth, and chromosomal male (46,XY) infants usually presenting in the first few weeks of life with a potential life-threatening adrenal crisis with salt-wasting, hypoglycaemia, and possibly cardiogenic shock (1, 2). More recently, the introduction of a newborn screening test for CAH, now done in over 40 countries, has the potential to diagnose infants prior to the onset of adrenal crisis (1).

CAH is associated with a number of additional clinical features that occur throughout an individual’s lifespan, some of which are associated with corticosteroids therapy. In children, clinical manifestations include growth impairment, short stature, and early puberty (2, 3). Adults are at risk of poor bone health, infertility, sexual dysfunction, obesity, metabolic disease, cardiovascular disease psychiatric disorders and alcohol misuse (2–5). In addition, there is an increased mortality in CAH associated with adrenal crises (1).

Management is difficult as overtreatment with corticosteroids can lead to growth failure or cushings syndrome, and undertreatment causes inadequate suppression of androgens, causing precocious puberty, advanced bone maturation, or virilisation (3). Increased doses of corticosteroids (and even hospitalization) are required for some common illnesses such as gastroenteritis to prevent life-threatening episodes of adrenal crises (1, 2).

In addition to lifetime medication with significant side effects, there are several controversial aspects in managing CAH. Early genital surgey in virilised chromosomal females (46,XX) is one of these controversies; ethical consideration such as a lack of informed consent from the infant themselves and poor long term outcomes with risks of impaired clitoral sensitivity, dyspareunia, and sexual dysfunction contribute to an argument to delaying non-essential surgery until the child is older and able to consent (1). Prenatal treatment with dexamethasone is also controversial; this therapy has been used for many decades to reduce virilisation of external genitalia in genetically female infants with classic CAH. In order to be effective, dexamethasone treatment needs to be started prior to confirmation of CAH diagnosis, thus risking unnecessary treatment of unaffected babies (6). Literature exploring the safety of prenatal dexamethasone is conflicting; prenatal dexamethasone has been associated with smaller birthweight, improved and worsened cognition, and differences in brain structure in individuals with CAH (6). In those without CAH, exposure to first trimester prenatal dexamethasone has been associated with brain structure changes and impaired pancreatic beta cell function, although there are no studies showing that this correlates to an increased risk of diabetes or cardiovascular disease (6, 7). Some studies have suggested negative impacts on cognition and problems with sociability in childhood in those without CAH (8, 9), although studies in adolescents and young adults have not been able to demonstrate differences in cognitive functioning (6, 10, 11).

As CAH is an autosomal recessive condition, mutation analysis is now able to identify the genetic abnormality in most cases, which has allowed a number of fertility interventions. While there are other forms of CAH caused by different gene mutations, 21-hydroxylase deficiency is the most common, causing 95% of CAH cases (3). Preimplantation testing (PGT) is a method where couples undergo *in vitro* fertilization (IVF) with genetic testing done prior to embryo transfer and implantation. All genetic and fertility-associated technologies are associated with unique challenges and their own controversies, including ethical or moral questions surrounding the value of life with and without genetic conditions (12). However, given the potential range of physical, psychological, social and ethical challenges, it is understandable that parents of children with CAH might value the option to avoid the birth of another child affected by CAH. As PGT allows for families to have an unaffected child without the possibility of needing to consider medical termination, it is often recommended to couples at risk of a pregnancy affected by a serious genetic condition (13, 14). Despite awareness of and availability of these assisted reproductive technologies, our anecdotal experience was that not all families choose to pursue these options, and others reject them after initial exploration or treatment failure.

While there are different types of PGT; for monogenic disorders such as CAH, only one gene is screened for (PGT-M). This paper will only be discussing the use of PGT-M so will use the acronym PGT for simplicity. An alternative to PGT, prenatal testing (PNT) after natural conception via chorionic villous sampling or amniocentesis, allows for subsequent management options including termination of affected pregnancies or antenatal dexamethasone to reduce virilization of affected females (14).

This paper documents the fertility choices of families in a single tertiary referral service (Sydney Children’s Hospital) of families with a child affected with CAH due to 21-hydroxylase deficiency, and explores their experiences and the fertility decision-making processes through qualitative, thematic analysis of semi-structured interviews.

The primary aims of this study were to explore fertility choices of couples with a child with CAH and the decision making process and perceptions behind these choices, and to explore the families’ lived experiences with CAH and the subsequent fertility journey. Ultimately, we aimed improve healthcare professional understanding of families in this situation to improve their ability to manage and support families with a child with CAH or other autosomal recessive conditions.

## 2 Methods

### 2.1 Participant selection and recruitment

Families with at least one child with 21 hydroxylase deficient CAH aged 0-18 years who were current patients of the endocrine department at Sydney Children’s Hospital in 2020 were eligible for the study. Clinical notes and family planning decisions of these families were reviewed by a member of the endocrine and study team to determine eligibility for interview. Families were invited to participate in a semi-structured interview if they had made an active decision to have or considered having further children.

As CAH is a genetic condition with autosomal recessive inheritance, it is routine, and considered best practice, for all families to be offered genetic counselling. Family planning genetic counselling refers to specific counselling in relation to future pregnancies and depends on the couple’s interest and readiness for this at the time.

### 2.2 Interview design

Semi-structured interview questions were written by a paediatric clinical psychologist (GB) with input from the research team. These questions are available as a **Supplementary Datasheet 1**. The same psychologist conducted the interviews. At the time of interviewing, he had over 30 years of clinical experience, a PhD in clinical psychology, and extensive research experience. Participants were aware of his affiliation with the endocrinology team. Some families had met him before the interview. There are no biases relevant to the research topic to disclose.

The interviews aimed to explore the parent’s experience with having a child with CAH, their experience with genetic counselling and genetic interventions (PGT or PNT), their family planning decision making process, and their overall impression of the health system. Interviews were conducted via an online video meeting platform (Pexip^®^). Interviews were recorded and transcripts were created. Interviews lasted approximately 60-90 minutes in duration. Participants were offered a copy of the transcripts after the interview.

### 2.3 Analysis

Interviews were independently listened to, and transcripts read by, two researchers using an inductive, semantic thematic analysis approach. The two researchers independently developed themes, then collaborated to refine the themes, which were critically appraised through regular meetings with the wider multidisciplinary study team. Themes and subthemes were coded manually and then refined as per Braun and Clarke’s framework (15).

Ethics approval and informed consent were obtained (Sydney Children’s Hospital Network Human Research Ethics Committee; 2020/ETH02974).

## 3 Results

### 3.1 Clinic and participant description

**Figure 1:**
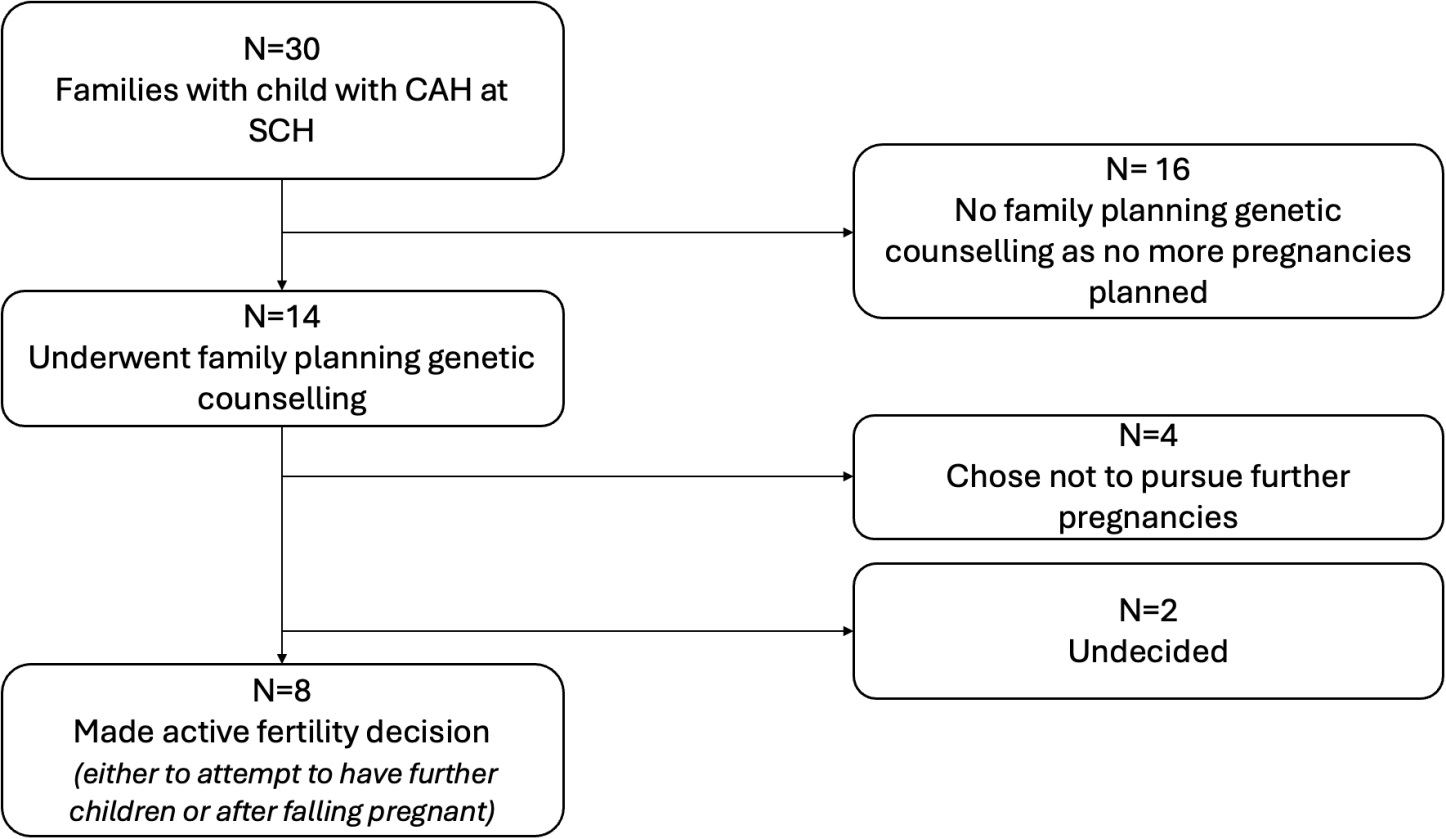
Overview of family planning choices of all families with a child with 21 hydroxylase deficient congenital adrenal hyperplasia. All 14 of the families considering other children or of a child-bearing age had received targeted advice from a geneticist, genetic counsellor, or endocrinologist regarding their options for family planning prior to falling pregnant. Of these 14 families, four did not pursue having further children after genetic counselling. Eight made an active fertility decision to have further children and are summarized in **Figure 2**. Two families have not yet conceived and have not chosen the path they would like to take. SCH, Sydney Children’s Hospital.

**Figure 2:**
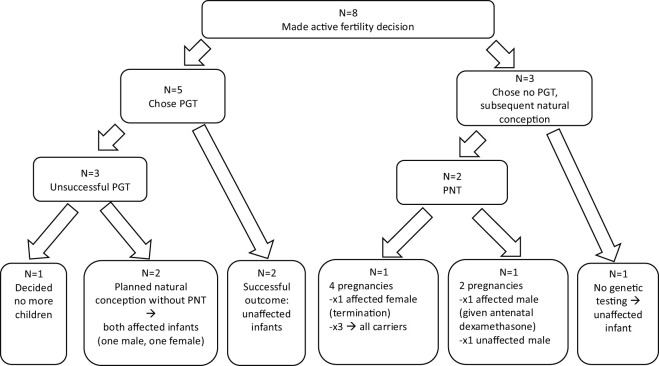
Summary of family fertility choices and outcomes for those who made an active decision to have another child after having a child with CAH. Five couples had preimplantation testing; two resulted in successful live births of healthy, unaffected, infants. One family, whose index child had a second genetic diagnosis, decided not to have any more children after experiencing PGT. Two families subsequently chose to conceive naturally with both going on to have affected children. Three families chose to undergo PNT after natural conception. Two of these had natural, unplanned pregnancies with affected girls and decided to terminate these pregnancies, with one of these going on to have three further infants all of whom were carriers. The third family had two planned natural pregnancies resulting in male infants (one affected, one unaffected). One family had an unplanned, natural conception and chose not to undergo any prenatal genetic intervention after genetic counselling and had an unaffected male infant.

At the time the study was conducted, the endocrine department looked after 34 children with CAH from 30 families. **Figure 1** shows the family planning decisions of these couples. Sixteen of the 30 parents declined family planning genetic counselling following their child’s CAH diagnosis as they had completed family planning and were not interested in specific family planning genetic advice. **Figure 2** describes the family planning decisions of those who made an active choice in having another child after their index child was diagnosed with CAH. Of these eight couples, all initially agreed to be interviewed, but one family was subsequently unable to be contacted. One couple was interviewed twice; once during pregnancy with their second child, who was conceived spontaneously without genetic investigations after “unsuccessful” PGT/IVF, and once 6 months after the female infant with CAH was born. Three families had both parents present for the interviews, while the rest of the interviews involved the mother of the children only. There was a broad spread of ethnicity, religion, education, ages and socio-economic status represented across the families interviewed, but this information is not included due to the potential risk of including identifying information.

### 3.2 Results: thematic analysis

There were six major theme categories identified which are summarized in **Table 1**.

**Table 1:** Summary of themes and subthemes identified in semi-structured interview qualitative analysis.

Theme Category	Theme	Subtheme	Quote
Impact of the index child’s CAH	Psychological impact of diagnosis	Profound and long lasting effects, causing symptoms of trauma Sex of child influencing experience Variability in coping mechanisms	“It was a dark time” “I did get depressed” “I think I had anxiety” “it was a little bit isolating” “[the way our male child was diagnosed] did create a lot of fear in us and took us a while to get to the point of having, even talking about, a second child” “So when we had [our child], we had already told people she was a boy, so that lead to a lot of confusion for people when we had to tell them that she was a girl” “I still get emotional … on his birthdays, because I think, oh we almost we might not be celebrating this and was so close to not celebrating it.” “I don’t know how women who don’t work. would have managed [to] psychologically get through it” “I find it confronting going into the hospital and being reminded of [the experience]” “It’s taken [my wife] quite a few years … to be able to talk about it without crying.”
	Negative impact on family unit, extended family and community	Impact on work and life choices Impact on relationships Isolation and secrecy	“We sort of left it in the family so really, no one outside our immediate family knows.” “It was very hard at the beginning … so I haven’t worked since she was born.” “We liked the idea of someone being home just in case the boys, just for emergency situations” “[Our parents] didn’t want us to have any more kids … because of the CAH” “[The way he was diagnosed] I think traumatised [my wife] and I and our parents for a while.” “I did feel. that I was in it completely alone”
	Longer term: reframing of disease impact over time	Neutral effect of CAH on day-to-day life Minimisation of index child’s phenotype Positive aspects of androgenisation Sibling bonding over shared condition Strengthening of parents’ relationships	“she was good at the things that girls were good at but she was also good at thing that boys were good at”“I think it’s actually, strangely enough, been a good thing for our relationship. We have learnt to trust each other more…” “Oh [our relationship is] probably better for it now.” “Even now when the boys are sick, we say to each other, ‘I can’t do this without you’ cause we are each other’s support. So it’s definitely had a positive effect [on our relationship].” “The fact that they’ve got each other … they both go for blood tests, they both go for doctors’ appointments, I think it helps them as well as us.” “He loves his CAH medication because it means he gets to go at lunchtime for his oral dose and gets to chat to the ladies in the office.”
Experience of Preimplantation Diagnosis	More burdensome than expected	Physical toll, medicalisation of process Emotional/psychological/social toll Financial cost Relationship strain Uncertainty	“It wasn’t a very pleasant experience, which of course, IVF isn’t going to be a [pleasant] experience, but it was very stressful. It was very. I know this is going to sound weird, but ‘clinical’” “It was very disappointing on every level” “I certainly think that whole process and decision making. it’s a very emotional. roller coaster”
	Outlook impacted by outcome	“Successful” outcome associated with a more positive outlook of PGT Unsuccessful outcomes associated with describing a more negative experience	“It was kind of a process, just a medical process … I probably felt more positive about it cause I felt like we had done what we needed to do to ensure that we didn’t have a child with CAH … so, the whole experience to me was more positive” “to the point where I was just like, this is not what it was sold, this was not what I expected, and for me there was just so many negatives, with no kid”
Family planning decision making	Dynamic nature of family planning decision making	Influence of lived experience of CAH Influence of lived experience of PGT Emotional versus intellectual decision making	“I don’t know whether it was something we ‘had to do’ get to the point that we were okay and were rolling the dice for the second time” “I think in the early stages we were both pretty fixed on the idea of not rolling the dice” “I think we had to go through that process [PGT] to get to the fact that we were comfortable [to conceive naturally].” “We had to wait to see [index child] grow up to realise that, this isn’t the end of the world.” “We were just saying that, the balance … with the IVF … the … emotional and … physical toll it’s taken [on my wife]. The length of time it was taking. The continued failures. And then at the end of the whole process you’re then having to make a decision about these mosaics, and you know, failed implantation … the pendulum really shifted for me [and my wife].” “As the IVF process went on, we went well hang on. Look, we don’t necessarily need to go down the IVF route to.” “So if it’s an affected boy I was still going to continue with the pregnancy because I already had 1 with CAH and I had already been 7 years into it and it is not as scary as it might sound.”
	Fear of the unknown	Sex of index child impacted fear Fear of a worse phenotype	“you don’t know that another kid wouldn’t have been as lucky” “So I am much more fearful of having a CAH girl … I think it will be a much tougher experience, in a different way…. Or longer haul … [The extended family] see [my child] and there is no fear of CAH now at least” “It scares me thinking that it would be born with half a vagina and half a penis or whatever and she won’t be able to have a normal pregnancy throughout her life and she will have too many male hormones and facial hair. I just can’t deal with that. It is not in my cards.” “The fact that [our first affected child] was a boy, and we knew we were having a boy, there was less fear in what was going to happen because we had already had a boy with CAH, so we kind of knew what was in store. Whereas, if we were having a girl with CAH, we were more concerned about what that could. Yeah, there’s all the other issues that go along with it with a CAH little girl”
	Accountability	Accountability to the future child Accountability to the index child	“We didn’t think that it was fair, knowing what we knew, that, why would we bring a child into the world, who then has this lifelong burden that we could have avoided.” “if we tried it and we had another child, or a girl, we could, that we could then tell them that had, you know, we had tried” “but if you went into it and you took that risk for somebody else’s life, lifetime issues, it just didn’t seem fair to me.” “we would have lost out on our time with [our index child].”
	Influence of religion/belief system	Genetic conditions affecting marriageability Religion objection to PGT or termination of pregnancy	“In our culture…… to get something genetic … they get a bit scared of things like that.” “It is a religious thing as well … not allowed to [terminate pregnancies]” “We come from a Christian background so we kind of think, or have the mindset, that these are the boys that we were meant to have” “It turned out to be a girl that has CAH. And we didn’t want to go down that path and we had to ask religious scholars if it was OK to terminate the pregnancy. And that was acceptable. Knowing they approved like I won’t be judged when I die that I terminated that’s one thing.” “Yep, but I am pretty sure in our Religion we can’t do that implantation pregnancy … Yea but I am pretty sure we can’t pick and choose if we want a child.”
Role of the healthcare professional	Role of healthcare professional in the CAH journey	Lack of understanding of CAH and exaggeration of life-threatening nature of disease Delay in specialist review at diagnosis contributing to distress Exaggerated trust of specialist medical professionals	“I was at that point where I just implicitly trusted anyone medical … after my son had been saved” ‘They couldn’t tell us what her condition was … No one really knew, the nurses had never heard of it. “They took the name off but they wouldn’t put anything back on, which was also kind of worrying”. We didn’t know if that meant maybe, what she had was fatal.
	Role of healthcare professional in fertility journey	Pressure to undergo PGT and judgement for choosing different options Variable experience with family planning genetic counselling	“I felt a lot of pressure [from the HCP] to do another round of IVF. There wasn’t, we kind of talked about, oh maybe we might be thinking of going naturally, and that wasn’t even ‘talked’ about. It was kind of just brushed over.” “It’s all very hard to understand but we kind of felt like we were being lead down the track of [PGT]”
Role of peer support in CAH and fertility journey	Influence and benefit of peer support	Universal positive experience of peer support Early peer support helped relieve distress Peer support gave perspective Providing peer support may be beneficial Peer support influence on family planning decisions	“I’ve gone down to meet them and it was lovely to see that she had a very normal looking child who was running around like you could imagine any toddler to do” “I don’t think it’s right to have a relationship with someone based on your children having a joint disability” “I spoke to a lot of people on the support group as well. A lot of parents that had two kids with CAH, so that helped get to that decision as well”
Experience with subsequent childbearing	Easier journey with subsequent child, regardless of CAH		“so it … felt more like a celebration where, when [index child] was born, it didn’t have that feeling” “And I did reflect on that when I had my second daughter, the difference between the way that I felt and the difference of the mood”
	Experience with second affected child with CAH	Unexpected grief and judgement with 2^nd^ affected child	“even though we knew it was a possibility that you’re going to have CAH, when we were told that he did have it, we were quite shocked” “because I felt like every time they came in, they were like, ‘why are you worried, you knew this was, you knew this was a probability, or a possibility’ like ‘why are you even upset”

#### 3.2.1 Impact of index child’s congenital adrenal hyperplasia

##### 3.2.1.1 Psychological impact of diagnosis

For all families, the psychological impact of their index child’s diagnosis of CAH was profound and long lasting. Some described symptoms of trauma, post-traumatic stress and depression, including low mood, anxiety, hyper-vigilance, and intrusive memories. For most families, the diagnosis of CAH occurred many years ago, yet this experience still had a psychological impact. Parents reported that these symptoms were exacerbated by feelings of isolation from family, friends, or their partner. Some families felt that the psychological distress led to a delay in their second pregnancy (of up to 8 years).

A family’s experience was impacted by the sex of their index child. In families with an affected female child, virilization of female infants led to confusion, distress and isolation from family and friends shortly after birth. All index male children in this study were born prior to the inclusion of CAH on NSW newborn screening, and as such, were often diagnosed after life-threatening adrenal crisis in infancy, causing an emotional response based on fear of death.

Coping mechanisms were variable. Some found work and distraction useful, while others thought the distress faded over time with lived experience with their child. Two families described the therapeutic effect of this study interview itself and felt that talking about their experience was beneficial.

##### 3.2.1.2 Negative impact on family unit, extended family, and community

All couples reported that their child’s CAH impacted their lives to some extent; including their ability to work, where they lived, and relationships with extended family and their community.

Uncertainty around gender assignment in virilized girls led to delays in announcing their child’s birth and subsequent negative reactions and perceived isolation from friends. Many reported keeping the diagnosis a secret from family, friends, and their cultural community, and that this contributed to social isolation.

Families often reported relationship strains with close relatives such as grandparents, the reasons for which included grandparent distress at the diagnosis, or a poor understanding of CAH by grandparents limiting their ability to support the family.

Couples also reported adjusting their professional careers; either to be closer to school or home in the case of an emergency or due to a lack of support or understanding in the workplace. Some parents had not worked since their children were born in order to be available at home in case they were needed. Others made decisions on where to live based on geographical proximity to specialist centers.

##### 3.2.1.3 Longer term impact of CAH: reframing of impact of disease over time

Parents reported experiences reframing their perceptions of the disease impact over time. All families found that the day-to-day burden of disease became more manageable with time and that this experience differed from expectations formed after discussion with healthcare professionals (HCP) around the time of diagnosis. The neutral impact of CAH on daily life, meaning that while the CAH was present in their daily life this change was neither negative or positive, was emphasized by all families. Families described a feeling that they were better off than other affected children, even when clinically it appeared that their child had a more severe phenotype. They also reported that the impact was minimal or less than expected; compared to other diseases or disabilities, such as head injury, epilepsy, or diabetes, CAH was not a significant burden. One family whose index child had a second, non-life-threatening genetic diagnosis reported that this second diagnosis was more stressful and burdensome than the CAH itself.

Not only was living with CAH less difficult than families had initially expected, but families often reported what they perceived to be benefits. For girls with CAH, many families verbalized positive aspects of androgenization; they reported that girls with CAH were often sportier and more able to relate to male friends than their female peers. For those with two children with CAH, the special relationship and similarities between them were presented in a positive light by parents. Similarly, some parents reported an overall positive impact on their relationship as a couple; the experience and supporting each other brought them closer together and increased trust in their relationship.

#### 3.2.2 Preimplantation diagnosis

##### 3.2.2.1 Pre-implantation diagnosis: a bigger (physical, emotional, social, and financial) toll than expected

All families reported a greater than expected physical, emotional, social and/or financial toll of IVF and PGT. Parents described a stressful or difficult time emotionally and often expressed surprise at the physical burden of the process, especially for the mother going through the IVF treatment. Some reported that it negatively affected their relationship with their partner. One major factor that most families emphasized, especially those for whom PGT was not “successful” (here defined as PGT and IVF resulting in the birth of a child with CAH), was the medicalization of a natural process. The uncertainty and subsequent difficult and complex medical decisions, such as implanting an embryo with a genetic abnormality of unknown significance, further led to a sentiment in some that this process was not worth the outcome.

##### 3.2.2.2 Outlook of PGT impacted by outcome

Families who had “successful” outcomes had a more positive outlook when recalling details of their experiences. Conversely, those who had “unsuccessful” outcomes reported a more negative overall experience of PGT and emphasized that this experience was different from expectations.

#### 3.2.3 Family planning decision making

##### 3.2.3.1 Dynamic nature of family planning decision making

A major theme identified throughout the interviews was how dynamic and complex the decision-making process was for families. Often choices changed over time and oscillated between options depending on current life circumstances, including lived experience of CAH with their index child. One couple described a shifting of their opinion regularly depending on whether their index child was well or unwell in hospital at the time. Similarly, the toll of PGT and repeated failures also influenced some couples’ decision to subsequently choose natural conception. Many couples described both emotional and intellectual aspects of decision-making; emotionally, the journey of experiencing PGT shifted “the pendulum” and allowed them to consider natural conception. For example, one couple stated that they had to “go through [PGT] to get to the fact that we were comfortable [to conceive naturally]”, and another felt their lived experience with CAH made risking a second child with the same diagnosis “not as scary as it might sound”.

The lived experience and realization of families of the low or even positive impact of CAH on the index child had a significant influence on family planning decision making. As the reality of day-to-day life with CAH became less frightening and overwhelming, and possible benefits of having another child with CAH (e.g. closer sibling relationships) became apparent, families felt that it became harder to justify why they would not want another affected child.

For some, the “unsuccessful” PGT experience was an important part of their decision-making process. Those who went on to conceive naturally expressed that they would not have done so without the PGT experience, and so did not regret PGT despite their negative experience.

##### 3.2.3.2 Fear of the unknown

One reason for all families who wished to avoid having a subsequent child with CAH was the fear of the unknown. Fear of the unknown was not only a fear of a worse phenotype but was also greatly influenced by sex of index child. Families whose index child was male feared the additional challenges associated with virilization in a girl with CAH, however felt after having lived with their sons’ CAH for many years felt that another boy with CAH would be manageable. This led one couple to decide to undergo PNT via CVS and terminate female affected infants, but not an unaffected, carrier, or male infant. For another couple, knowing their naturally conceived child was a boy, even though they did not know if they were affected, was reassuring, and helped reduce stress levels during pregnancy. In addition to clinical phenotype, child personality traits and resilience was discussed; one parent highlighted the social, outgoing nature of her child as an important factor for him dealing well with this condition. Therefore, having another affected child with less resilience was also a concern for some families.

##### 3.2.3.3 Accountability to their future child and index child.

Accountability to their future child was an important factor for many families in their decision to pursue prenatal genetic intervention and avoid a diagnosis of CAH. Some described this as related to guilt about their current child’s diagnosis, the fact that they had passed on the genes, or parental responsibility to try to prevent their child having a condition with lifetime implications.

Others felt that the PGT process itself, even when it was “unsuccessful” and they then went on to conceive naturally, allowed them to be accountable to their future child as they had tried all options. Some parents reported concern that having another child with CAH was unfair to the index child as it would take time and energy away from them, and so chose not to have another child after “unsuccessful” PGT.

##### 3.2.3.4 Influence of religion, ethics, belief system

Religion, ethics, belief system and culture also had an influence on a couple’s fertility choices as well as their CAH and fertility journey. Religious or ethical beliefs that impacted on fertility decision making included rejection of PGT and/or termination based on religious teachings, and a belief in pre-determined fate. Religion had variable influences; even within the same religion, there were differing opinions on whether termination or PGT were acceptable. One parent also described that the cultural stigma against genetic conditions, in particular in relation to marriageability, contributed to their initial grief after diagnosis and concern not only about having another child with CAH but also about another child being a carrier. Several families have been unable to share their child’s diagnosis and/or IVF/PGT journey with extended family, friends or community due to their own or others religious beliefs.

#### 3.2.4 Role of healthcare professional

##### 3.2.4.1 Role of healthcare professional in CAH and fertility choice journey

Healthcare professionals (HCP) had an important role throughout the CAH journey for families. At the time of initial diagnosis for their index child, a lack of understanding and exaggeration of the life-threatening nature of disease often contributed to psychological distress. Additionally, prolonged delays and wait times to see the endocrine specialist contributed to confusion and distress. Some families felt that seeing the endocrinologist earlier would have led to a less traumatic experience, as they felt that meeting the endocrinology team was reassuring and helpful.

All families described positive experience with their specialist endocrine healthcare team. For some, this close relationship led to high expectations and often exaggerated trust of the healthcare team. For one parent, after her index child had been diagnosed and their life “saved”, she described an “implicit” trust of medical people and was disappointed when she felt she was mismanaged during her IVF journey; she felt that her inability to question this may have contributed to the “unsuccessful” outcome.

Families often felt that HCPs, especially fertility specialists, were biased in presenting options for conception, and felt pressured to undergo PGT and judgement if they opted for a different pathway such as natural conception. One family stated that the option of conceiving naturally “wasn’t even talking about … it was kind of just brushed over”. Similarly, those who naturally conceived a second affected child felt judgement from HCPs, both pre and post conception, including from postnatal midwives who did not understand their shock or grief at a second child’s CAH diagnosis as they “knew [another diagnosis of CAH] was a probability or possibility”.

#### 3.2.5 Role of peer support in CAH and fertility journey

##### 3.2.5.1 Influence and benefit of peer support

All families who had received peer support within the CAH community reported a positive experience. Peer support in the early days after diagnosis helped to give hope to families and thus eased psychological impact. It also provided perspective for families; seeing more severely impacted individuals with CAH reinforced their impression that their child was mildly affected and that they were better off than others. Social media or online forums were also mentioned by some families and sometimes influenced their family planning choices. Peer support did not have to be condition specific; one parent reported that she did not see any benefits from peer support within the CAH community, although did describe seeking and benefiting from companionship from parents of children with other disabilities or conditions. Families asked to provide peer support reported feeling validated they were managing well, and had a positive experience in supporting others.

#### 3.2.6 Experience with subsequent childbearing

##### 3.2.6.1 Experience with second affected child with CAH

All families reported that the journey with their subsequent child was much easier than their experience with their index child, regardless of whether or not they were affected with CAH. Some reported that the birth of their subsequent child was a positive experience, compared to the negative, stressful experience with the index child. Newborn testing leading to earlier diagnosis was discussed as another positive factor influencing the experience of CAH diagnosis. Others reported distress after experiencing unexpected grief and judgement with their second affected child. Even though it was a known possibility that their child could be affected by CAH, parents reported feeling shocked and upset by the news, and this was compounded by a lack of empathy or understanding from some HCPs surrounding them after birth

## 4 Discussion

We aimed to explore the fertility choices of couples with a child with CAH and the decision making process and perceptions behind these choices, and to explore the families’ lived experiences with CAH and the subsequent fertility journey. Our hope was that an improved HCP understanding of families in this situation may improve their ability to manage and support families with a child with CAH or other autosomal recessive conditions. This study identified the influence of a wide variety of factors that contribute to a family’s CAH and fertility journey; including a family’s social environment, education, religious and cultural beliefs, the evolving experience with their index child, the health system and HCPs, and peer support. This study highlights the complexity and dynamic nature of family planning decision making, and the HCP’s awareness of this when family planning options and choices are discussed.

This study highlights how complex and dynamic the fertility decision making process is for couples with a child with CAH. Many of the couples’ perspectives and choices around future children changed or oscillated between options over time, often related to the unexpectedly increased toll of PGT along with their lived experience of CAH in their index child. The dynamic nature of family planning decision making has been previously described in families with children with other genetic conditions (13, 16). Qualitative research looking at couples at risk of having a child with genetic conditions have found that couples may spend a long time contemplating PGT (up to 36 months) (16), and that once they do make a choice, this decision may change due to practical, economic or emotional reasons (16, 17).

The IVF and PGT experience for couples in our study was more burdensome than they had anticipated; physically, emotionally, psychologically, financially and from a relationship point of view. This is also consistent with previous research; studies have found that those undergoing PGT not only find it a practical and logistically difficult process, but also stressful and psychologically demanding, associated with emotions such as uncertainty, grief, helplessness, anxiety and depression (13, 18–22). Our study was consistent with the literature that the overall burden of IVF and PGT is particularly high for women, and that the psychological impact of PGT can be long lasting for both parents (22). Interestingly, despite this greater than expected toll on couples, none of the individuals we interviewed expressed regret at their decision to undergo PGT, including those with a negative outcome. This is also consistent with the wider literature in PGT regardless of outcome or indication (18, 19, 22–24).

Couples included in this study, all of whom had previously had spontaneous pregnancies, were often surprised when IVF was “unsuccessful”. Studies show that there are elevated expectations of success with resultant increased disappointment after “unsuccessful” cycles in couples with normal fertility undergoing IVF and PGT (13, 23, 25). Higher levels of awareness of PGT limitations have been shown to result in more positive experience, greater trust, and better relationships with clinical staff (17). A study based out of the USA found that couples often placed too much trust in their HCP, consistent with the theme our study identified of an exaggerated trust in the healthcare team (26). Families in our study often felt that HCPs, especially fertility specialists, were biased in presenting options for conception, often not even acknowledging or discussing the option of natural conception or any other alternatives. This may be due to a poor understanding of CAH and not appreciating the improved quality of life when it is well managed, a lack of appreciation of the burden of PGT for some couples, along with possibly their own need to be offering an active treatment. Couples therefore felt pressured to undergo PGT and judgement when they opted for a different pathway such as natural conception, even after “unsuccessful” cycles. Therefore, we suggest that managing expectations of couples undergoing PGT and an open minded and empathetic approach to family planning genetic counselling may mitigate the psychological distress associated with “unsuccessful” cycles.

In our study, the major themes impacting on family planning decision making were accountability to the future child, fear of the unknown, and lived experience with PGT and CAH, with financial aspects and belief systems also significant considerations. Primary factors identified to influence fertility decision making varies across the literature. In making decisions around PGT or PNT, couples consider many aspects; religious or ethical/moral (especially beliefs surrounding termination of pregnancy), media, financial, understanding of the condition being tested for, and feelings of responsibility (to avoid having an affected child) (18, 23–25, 27, 28). Studies also suggest that reproductive history, such as history of failed IVF, as an important factor in decision making, and an even more important predictor for using PGT than the clinical impact of the disorder being tested for (18, 29).

Accountability to the future child was an important factor particularly for those who decided to undergo PGT. Interestingly, even those who went on to have a naturally conceived second affected child after “unsuccessful” PGT, felt that the experience of PGT itself not only helped them make the decision to, in their words, “roll the dice” on natural conception, but also allowed them confidence that they had done all they could to avoid their future child’s disease. This feeling of accountability regardless of outcome was likely a contributing factor in not regretting PGT despite the negative experience, a finding that is also consistent with other research (18, 19, 22–24). However, not all families felt this way; one couple opted not to have any more children after “unsuccessful” PGT because of a perception that the process would have been wasted if they had then gone on to conceive naturally.

Fear of the unknown was a driving factor behind fertility choices. This was greatly influenced by sex of index child. This has not been identified in the literature to date and is an important finding of this study. Families feared a worse or different phenotype, such as more severe cortisol deficiency or a virilized female, in a different child who may have less protective qualities such as resilience or confidence. This fear of the unknown led couples to make specific choices, such as in one case terminating pregnancies with affected female infants; it is unclear if addressing these fears through HCP counselling or peer support may change these decisions. Understanding that a fear of the unknown or “worse” situation may be a driving factor behind fertility decisions is important for clinicians to keep in mind when counselling families regarding their options, and when considering resources such as peer support, to ensure couples make fully informed decisions.

Religious, ethical or moral belief systems were another recurrent theme identified that influenced the decision-making process, as has been documented in the literature (13, 16, 21, 30), although the choices made even for those with the same religion were not consistent. While some reported that their religious or moral belief systems would not allow PGT, for others this was preferable to termination of pregnancy after PNT. Religion not only impacted on the choices made but also the psychological impact of a CAH diagnosis and, for some, contributed to isolation from friends, family, and community.

In addition to the fertility decision making process and family planning decision, this study explored the lived experience of CAH and how this in turn impacted their fertility journey. Our results emphasise that the psychological impact of a child’s diagnosis of CAH for parents can be profound and long lasting, and that it may be influenced by HCPs. This is consistent with other research looking at CAH and disorders of sexual development (DSD), where symptoms of post-traumatic stress were commonly seen (31). This psychological distress complicates family planning decision making and may lead to delaying plans for subsequent children and drive couples to consider options to avoid having a second child with CAH. The grief couples feel at diagnosis may be worsened by misunderstanding, miscommunication, and a lack of empathy from HCPs. In these interviews, couples expressed that, in retrospect, the seriousness of the disease was overemphasized by HCPs at the time of diagnosis. They felt that review by specialist endocrinologists helped relieve the distress and allay their fears, with many suggesting that earlier review may have improved their experience. These findings are consistent with literature on rare diseases; a systemic review of parent experiences looking at rare congenital disorders found that families may be frustrated when HCPs do not understand their child’s care needs, and overall lack of understanding of their child’s condition led to feelings of anger, frustration, or isolation (32). This review found that factors promoting positive experiences for families included engaged and understanding HCPs and social support (32). We suggest that a family’s CAH journey may be improved by earlier endocrine specialist review, education and training of postnatal ward staff on CAH, and an increased awareness by HCPs of the depth of the psychological impact of the diagnosis on families and their own contribution to this distress. Overall, a multidisciplinary approach with shared decision making between families and the involved specialties, and early psychological counselling at the time or shortly after CAH diagnosis, is recommended.

Long-term, couples reported that the psychological impact of the diagnosis was lessened by time and positive or neutral lived experience with CAH. Over time, families reported that the daily impact of disease was minimal, and that there were benefits of having a child with CAH. All parents expressed gratitude that their child was healthy and relatively normal, and most expressed a feeling that the seriousness of CAH was oversold by HCPs early on. Interestingly, this mindset was universal and not related to severity of clinical phenotype. This lived experience of CAH then often caused a shift in mindset for couples considering a natural pregnancy, “risking” having a subsequent child with CAH. Perceptions of “seriousness” by individuals living with genetic conditions have been previously explored in a large survey and qualitative interview study in the United Kingdom of multiple “clinically significant” diseases (including haemophilia, thalassaemia, spinal muscular atrophy, cystic fibrosis, and Fragile X syndrome) (33). This study found similarly that lived experience of a disorder, as assessed by self-reports of good health and quality of life, was better than one would expect from a “clinically serious” condition. This was most likely to be the case in those diagnosed earlier in life, as their condition becomes incorporated into their personal identity. In addition, very few individuals supported selective pregnancy termination for their own condition. Another review study looking at quality of life in 30 rare genetic conditions found that quality of life was not necessarily lower in affected individuals compared to their unaffected counterparts, which they hypothesized was due to a response shift, that is a reframing and adjustment of expectations, as was seen in our cohort (34). This suggests to HCPs who are counselling families early on, that emphasizing the seriousness of CAH may not only lead to increased distress at a difficult time for families, but may not be an accurate prediction representation of their future experience. Of course, as these studies are based in high income countries such as Australia, the experience of those in lower income countries with reduced access to healthcare may be very different, and therefore local perspectives should be considered.

One way to ensure families understand what life may look like with a rare disease is through peer support. Our findings emphasise the potential benefits of peer support in supporting families with CAH. Meeting other families, through peer support, helps families to develop realistic expectations for what to expect in the future. Social and peer support has been shown to facilitate adaptation to chronic illness and improve the experience of families living with a child with a rare disorder (32, 35). In our study, families who experienced peer support, either through meeting families with a child with CAH, another chronic illness, and families who provided peer support themselves, reported a universally positive experience. Peer support has been shown to be effective across a wide variety of health problems and populations; one study looking specifically at families with a child with CAH, reported significant benefits in meeting other children with CAH and their families, including reduced feelings of isolation and stigmatisation (36). Due to these benefits, we suggest that peer support should be offered to all families of children with CAH from diagnosis and throughout their CAH and fertility journeys.

Social media and online resources were also frequently accessed by our families and had both positive and negative influences on fertility decisions and a family’s CAH journey. Social media validated some families’ decisions to pursue natural conception and strengthened others’ resolve to avoid a subsequent pregnancy affected by CAH. Social media use is very commonly utilized by those with and without chronic disease and is often used to access medical information or peer support (37). The literature reports a variable benefit of social media use in chronic disease; a recent review reported that 48% studies indicated a benefit of social media for individuals with a chronic illness, 45% neutral or undefined, and 7% suggested harm (38). Other studies have described how social media can be used to better support disease management and psychological support and that online engagement may correlate with lower self-reports of stress and depression (38–40). Nevertheless, HCP awareness of the influence of online information is important in understanding a patient and family’s decision making and journey, and it may be helpful to pre-emptively supply families with reliable sources of information and support.

This is the first study to look at PGT and fertility decision making for couples at risk of CAH and describes some important considerations for clinicians wishing to improve the care of these families. While this study represented a relatively small cohort from a single site, the rate of uptake of interviews was high (seven out of eight eligible families were interviewed). The multidisciplinary specialist team and immense experience of the interviewing psychologist are strengths to this study. Limitations of this study include the small sample size, a common issue with rare disease research, and the single centre experience. It would have been valuable to also interview those who did not consider further children to investigate the reasons behind these processes and to compare the experiences of these families to those who did wish to have more children. In addition, only three of the families interviewed had both parents present, with the others involving only the mother of the child in the interview. This was due to participant choice and practical reasons, but we acknowledge that interviewing both parents may have led to additional insights.

Further research including larger cohorts from different cultural backgrounds, in different centers and countries, would be valuable as CAH and PGT/family planning journeys are likely to vary. For example, the financial toll on couples varies greatly between countries. In particular, studies from the USA show that cost weighs heavily into couple’s decision-making process when it comes to IVF with PGT, as compared with Sweden or Hong Kong (13). In Australia recently, since our study was conducted, government rebates have been introduced for PGT for carriers of CAH among other disorders, a result of advocacy after a research study screening program called MacKenzie’s Mission (41). This will likely have an impact on families in the future, although the IVF costs themselves are not usually covered. Whilst newborn screening reduces the risk of adrenal crisis and likely will change the journey of families, preliminary research has found it does not completely mitigate the distress (36). More research on the impact of newborn screening on future family planning on a family’s journey with CAH is needed.

## 5 Conclusions

This study explores the family planning decision making process of couples with CAH, as well as their CAH and fertility journeys. These data highlight some important considerations for HCPs looking after families of children with CAH. Firstly, a greater understanding of the complex and dynamic nature of family planning decisions will hopefully allow HCPs to better support couples and improve the overall experience for these families. Additionally, HCPs should keep an open mind and maintain empathy when counselling couples on fertility options. In particular, the HCP awareness of the psychosocial and physical toll of PGT and its limitations, and communication of this to couples to manage expectations, is important. In regards to managing a child with CAH and their family, HCPs should be aware of the depth of the psychological impact of the diagnosis on families and our own contribution to this. The initial distress surrounding diagnosis may be improved by better communication, education, and earlier specialist review. While the journey with a second child (with or without CAH) is usually better for parents, couples still require emphathy and non-judgemental care at this time. Offering and encouraging peer support throughout a family’s CAH and family planning journey may also be beneficial. Future research would be valuable in expanding this research to different populations, cultures and healthcare systems.

## Data Availability

The original contributions presented in the study are included in the article/**Supplementary Material**. Further inquiries can be directed to the corresponding authors.
