# Supplementary material for: Family planning and preimplantation testing: family experiences in congenital adrenal hyperplasia
Source: Front Endocrinol (Lausanne). 2025 Jan 7;15:1482902. doi: 10.3389/fendo.2024.1482902 (PMC11746090; doi:10.3389/fendo.2024.1482902)
Supplement: Supplementary file 1 [file DataSheet1.docx]

| **Study Information** | |
| --- | --- |
| **Title** | Family Planning in Congenital Adrenal Hyperplasia: exploring the experience for families |
| **Semi-structured interview structure and questions** | |

Confirm with parent/s for each interview their family genogram, diagnosis and medical interventions used for each pregnancy and/or child, and where they are up to in their family planning (finished or planning/considering other pregnancies).

A. Experiences of Congenital Adrenal Hyperplasia (CAH)

1. I understand one (or more) of your children has CAH? How are they progressing medically/socially/academically/psychologically &c? Are you having (or did you have) difficulties in managing your child or your child’s CAH? How is your child coping with CAH? Do you feel you have enough support in managing your child with CAH? Could there be any improvements in the medical/childcare/school &c systems to help?
2. Was the experience of having your current child with CAH different/same as you expected? How? Tell us about the history of your pregnancy with this child and about your experiences following the child’s birth. This includes the effects on (and reactions of) your child, yourself, your partner, your children, your family, your friends, and anyone else significantly involved in the child’s life (e.g. doctors, or medical/nursing staff). How has the birth of your child with CAH affected you and your family’s life? Could this period of your life have been assisted or made easier by anything (e.g. changes in the medical or other systems)?
3. How are you and your immediate/extended family managing now with your child with CAH? Are there any issues of concern? How are your other children reacting?
4. What were your experiences during and after your current child’s birth? Did things go smoothly or where there medical or emotional issues?
5. Do you feel you experienced any adverse emotional reactions to the birth beyond the normal stressors mothers/parents face during/after the birth? E.g sadness, anxiety, anger, depression, &c)
6. Given your child’s diagnosis (whether unaffected or affected), what were your reactions when told? How did the process of diagnosis go for you and your family? Based upon all you have experienced, if you had your time again, would you have made the same decisions with respect to the pregnancy?
7. Given your child’s circumstances, what treatment will he/she require, will you seek?
8. Tell us about the process you anticipate of bringing your child up (where child has CAH) and your philosophy around this? E.g. will you and when will you inform them about CAH? When will you get them involved in helping to make decisions about their future (e.g. surgery, in cases of ambiguous genitalia) &c?
9. Do you feel you have a sound knowledge about CAH? Can we discuss the basics of what you understand about CAH (origins, symptoms, treatment, challenges, risks, &c). Where did you get information about CAH from? Do you feel what you were told and how you learned about CAH was adequate? Could the system be improved? How?
10. Do you or your family have a previous history of CAH (and/or other genetic/medical conditions)? Can you tell us about your experiences (e.g. feelings about/reactions/difficulties), beliefs/philosophy, psychological approaches, &c in managing such conditions?
11. Having discussed the diagnosis and treatment and experiences surrounding your child’s CAH have you any further suggestions for the health (or other) systems about dealing with people going through the process of diagnosis and treatment that your child and you as parents went through?

B. Experiences of Genetic Counselling

(1) Following the birth of your child with CAH, were you offered any kinds of family planning advice (e.g. genetic counselling, &c)? Please explain your understanding as to why family planning advice was offered to you in relation to the birth of your child with CAH?

(2) Can you recall what kind of family planning advice you received? From whom?

(3) Did you use the advice to guide you in your future planning? How? Are you satisfied with the choices you made or would you have made a different decision now based upon your experiences?

(4) If you did not use the advice to guide you, why not (i.e. what was your thinking process at that time)? What were your decision and actions based upon at that time (i.e. what do you think influenced you)? Thinking back now, are you satisfied with your choices in relation to this or would you now have done anything differently?

(5) Whether or not you applied the family planning advice you were given, do you think the advice provided was complete enough, and understandable enough, and presented adequately to guide you with regards to the next steps in planning your family? Can you indicate any ways in which this advice could be improved (e.g. the way it was delivered, content, &c)?

C. For those Parents who went on to Conceive Naturally

(1) You received family planning advice, and from it you were aware that it was possible that a future pregnancy could result in you having a child with CAH. Is this a correct statement of your experiences? If not, tell us more about this. At some stage, for one of your pregnancies, you decided to conceive naturally, without using other medical technology that would ensure you did not have another child with CAH. Can you tell us what influenced you in deciding to go ahead and conceive naturally, knowing what you knew then?

(2) Can you tell us about your experience with this naturally-conceived pregnancy? Did you go on to complete this pregnancy? If not, would we be able to discuss how the pregnancy ended? Tell us as much as you are comfortable to disclose about this situation and your experiences? Could anything have been done differently by anyone (e.g. staff, &c?)

(3) If the pregnancy was completed, was your child from this pregnancy affected by CAH? Whether or not your new baby was affected by CAH, we are interested in knowing how their birth affected you emotionally and psychologically? How did the birth affect your partner and immediate and extended family and friends &c? Were there any negative repercussions?

(3) Do you recall anyone expressing an opinion to you (before or after birth) about your choice to conceive naturally? Who? Tell us more about this and how it affected you or your partner or family and friends? For example, did you ever get an impression that anyone made a judgement (positive or negative) about your decision to conceive naturally? Who were they? What are your thoughts about them making this judgement? Do you feel you have experienced any repercussions from such an experience (positive or negative)? What sort of repercussions?

(2) In hindsight, can you think of anything you would do differently with this naturally-conceived pregnancy, knowing what you know now? Is there anything you could recommend to other involved they could do differently (e.g. staff, family, &c)?

D. For those Parents who used Preimplantation Testing

(1) You received family planning advice, and from it you were aware that it was possible that a future pregnancy could result in you having a child with CAH. Is this a correct statement of your experiences? If not, tell us more about this. At some stage, for at least one of your pregnancies, you decided to conceive using preimplantation testing to ensure you did not have another child with CAH. Can you tell us what influenced you in deciding to go ahead and try to conceive using preimplantation testing, knowing what you knew then? Did you feel the advice and treatment you received from staff at this time were satisfactory? Tell us more.

(2) Ultimately, following this preimplantation testing procedure, did you obtain viable embryos? Can you tell us more about this whole experience?

(3) Did you decide to go ahead and try to have one of these unaffected embryos implanted? Tell us about your personal experiences and reactions to preimplantation testing and with in-vitro fertilization.

(4) If you went ahead with implantation, was this pregnancy successfully completed? If not, tell us as much as you are comfortable to, about this situation and the effects upon you and those around you? Did this experience affect the planning of future pregnancies for you (for example did you try the same procedure again, decide not to try to get pregnant again, or did you try to conceive naturally)? Can you tell us as much about this and its effects upon you as you feel comfortable to?

(5) If the pregnancy with an unaffected embryo implanted was successful, tell us about your experience of this pregnancy, the birth and any consequences? For example, how did staff or family respond to you in relation to this? Did this experience affect your decisions with regard to planning future pregnancies?

(6) If you decided not to proceed with implanting one of these unaffected embryos can you tell us about how you came to this decision? What or who influenced you? How did people around you respond to this decision?

(7) If you decided not to proceed with implantation, did you consider (or did you actually) try to conceive naturally instead? Can you tell us about your thinking here? That is, why were you considering conceiving naturally at this stage or why did you proceed to try to conceive naturally? Did other people know about this decision or come to know? How did they react and what was the effect upon you and the people around you?

(8) Can you tell us about your experience with this pregnancy? Did you go on to complete this pregnancy? If not, would we be able to discuss how the pregnancy ended? Tell us as much as you are comfortable to disclose about this situation and your experiences? Could anything have been done differently by anyone (e.g. staff, &c?)

(9) If the pregnancy was completed, was your child from this pregnancy affected by CAH? Whether or not your new baby was affected by CAH, we are interested in knowing how their birth affected you emotionally and psychologically? How did the birth affect your partner and immediate and extended family and friends &c? Were there any negative repercussions?

(10) Do you recall anyone expressing an opinion to you (before or after birth) about your choice to conceive naturally? Who? Tell us more about this and how it affected you or your partner or family and friends? For example, did you ever get an impression that anyone made a judgement (positive or negative) about your decision to conceive naturally? Who were they? What are your thoughts about them making this judgement? Do you feel you have experienced any repercussions from such an experience (positive or negative)? What sort of repercussions?

(11) In hindsight, can you think of anything you would do differently with this naturally-conceived pregnancy, knowing what you know now? Is there anything you could recommend to other involved they could do differently (e.g. staff, family, &c)?

E. For those Parents who used chorionic villus sampling (CVS)

(1) You received family planning advice, and from it you were aware that it was possible that a future pregnancy could result in you having a child with CAH. Is this a correct statement of your experiences? If not, tell us more about this? At some stage, for at least one of your pregnancies, you conceived (either naturally or with medical assistance) and used chorionic villus sampling (CVS) or another technology to determine if your foetus was affected by CAH. Can you tell us about your experiences here? Can you tell us what influenced you to take this approach, knowing what you knew at the time? Was this prior to the availability of preimplantation testing? If not, why did you decide one method over the other? Did you feel the advice and treatment you received from staff at this time were satisfactory? Tell us more.

(2) Whether or not you used CVS results for each pregnancy, was each pregnancy successfully completed? If not, tell us as much as you are comfortable to, about each situation and the effects upon you and those around you (for example, did the pregnancy end due to termination or miscarriage)? Can you tell us about how this decision or this situation came about? How did each of these experiences affect the planning of future pregnancies for you (for example did you try the same procedure or approach again, decide not to try to get pregnant again, or did you try to conceive naturally)? Can you tell us as much about this and its effects upon you as you feel comfortable to?

(3) Can you tell us what your experiences were with medical system and the effects on you and your family for each of these pregnancies? Did the way the staff or the system interacted with you and your family influence your decisions with regard to the pregnancy or future pregnancies or your family planning? Could you advise as to any improvements or changes that could be made to the system that would help you or future patients? How did your partner or family or your wider acquaintances react in relation to your experiences and decisions surrounding each pregnancy?

(4) In the case of each pregnancy completed, tell us about your experiences of this pregnancy, the birth and any consequences? Do you feel that how you had been interacted with by staff or others affected you (whether adversely or positively). Did this experience affect your decisions with regard to planning future pregnancies?

(5) In the case of any of these pregnancies where you got a CVS (or other) result suggesting or indicating your foetus was affected by CAH, can you tell us about your experience of this and the reactions of anyone else you informed around you? Did you get advice or comments from medical staff at this time? Did you decided to maintain the pregnancy or terminate your pregnancy and how did you come to this decision? Who or what influenced you at the time? Were the advice or comments of staff or others helpful for you or did they adversely affect you? Do you have any regrets about your decisions? Would you have done things differently, if you knew what you know now?

F. Overall Impressions of the Health System

(1) What was your experience of the health system? Where could it be improved? How? Did you feel anyone adversely influenced you, or was trying to influence you, when you had to make a decision about testing or treatment? Did you feel, for example, under any undue stress or pressure? Tell us as much about this as possible.

1. Do you have any suggestions for the health system about dealing with people going through the process you as parents were going through? Do you feel the system helped you to achieve what you wanted to with the pregnancy or pregnancies? Were you satisfied with how things went and the way staff managed you and your child’s situation?
